# Herpes Zoster Eruption in an Otherwise Healthy Child: A Case Report

**DOI:** 10.7759/cureus.5194

**Published:** 2019-07-22

**Authors:** Abdullah Ashi, Abdullah Ali, Mohammed Alzahrani, Jumanah Ali, Rawia Albar

**Affiliations:** 1 Pediatrics, King Abdulaziz Medical City, Ministry of National Guard Health Affairs, Jeddah, SAU

**Keywords:** varicella-zoster virus (vzv), reactivation, herpes zoster, child, shingles

## Abstract

Varicella-zoster virus (VZV; human herpesvirus 3) is a herpesvirus that causes infection in humans. The reactivation of latent VZV manifests as herpes zoster or shingles. In immunocompetent children, reactivation is rare, as increasing age is the most common risk factor for reactivation. The complications of infection include post-herpetic neuralgia and neurological sequelae as well as a bacterial infection of the crusted skin. We present a case of an otherwise healthy 13-year-old child with herpes zoster and multiple risk factors, who was managed successfully, in order to expand the limited literature. The patient presented with a painful vesicular rash, which appeared as multiple grouped vesicles on an erythematous base spreading over the right half of the face. The diagnosis of herpetic (varicella) vesiculobullous dermatitis was confirmed by biopsy and the patient was started on acyclovir and clindamycin with gradual improvement and resolution of his symptoms. Reactivation of VZV is considered a consequence of decreased cell-mediated immunity. However, the reason for reactivation in immunocompetent children remains unclear. In the present case, the patient may have become exposed in utero, through vaccination, or as a result of major facial trauma sustained at the age of six years.

## Introduction

Varicella-zoster virus (VZV; human herpesvirus 3) is a human herpesvirus infection that manifests in two clinically distinct forms: primary varicella and herpes zoster. As the primary varicella infection resolves, the virus passes through sensory neurological tracts of the affected area, eventually residing in the dorsal root ganglion of the infected dermatome in a latent form. Reactivation of the latent virus manifests as herpes zoster, which is commonly known as shingles [1]. Clinically, herpes zoster is described as a multivesicular eruptive rash that follows a single or multiple adjacent dermatomal distribution. It is often accompanied or preceded by acute pain and itchiness. Complications of the infection may arise, such as post-herpetic neuralgia, a bacterial infection of the crusted skin, and neurological complications [2]. These complications can lead to impaired quality of life and even functional disabilities. Reactivation in children is rare, as increasing age is the primary risk factor in the general population [3]. We present a case of a 13-year-old child, who was otherwise healthy, with herpes zoster with multiple risk factors, including intrauterine exposure to VZV infection.

## Case presentation

A 13-year-old boy, not known to have any medical illness, presented to the emergency department of King Abdulaziz Medical City in Jeddah, Saudi Arabia. The patient complained of a painful, itchy, vesicular rash affecting the right side of his face. The pain was severe enough to prevent him from opening his mouth and eye. Three days prior to presentation, he had started to experience a burning pain described as a toothache at the right upper angle of his mouth. One day after the pain started, he developed a vesicular rash affecting the right side of the oral mucosa and spreading progressively to the right side of the nose, reaching the right lower eyelid and causing redness of the sclera. On presentation, blood tests revealed an elevated level of C-reactive protein of 9.4 mg/L and a white blood cell count of 3.9 × 10^9^/L. A swab culture from the face lesion was negative for herpes simplex virus (HSV) types 1 and 2. VZV-specific immunoglobulin G (IgG) was less than 10.0 g/L, with negative IgM. A skin punch biopsy was taken from a vesicle with a maximum diameter of 0.3 cm. The sections of skin showed intraepidermal vesicles with multinucleated cells and epidermal dyskeratosis with intranuclear smudged inclusions. The pathology report confirmed the diagnosis of herpetic (varicella) vesiculobullous dermatitis. A blood sample was drawn from the patient and sent for molecular testing (polymerase chain reaction (PCR)). A computed tomography (CT) scan of the head and neck showed normal brain parenchymal enhancement and right neck soft tissue swelling with no acute brain insult or lymph node involvement.

The patient was a full-term baby of a healthy mother. Apart from varicella infection in the mother around two weeks prior to delivery, the pregnancy was uncomplicated. Upon delivery, the baby was admitted to the neonatal intensive care unit for observation because of suspected neonatal varicella infection. Three days later, he had not shown any signs or symptoms of infection, had not received any treatment, and was discharged in good health. The child received VZV vaccination at the age of 18 months and six years, as part of the Saudi Arabian National Vaccination Program. The mother did not report any previous varicella infection. At the age of six years, he sustained major trauma to the face, with lip laceration and bleeding that required suturing.

**Figure 1:**
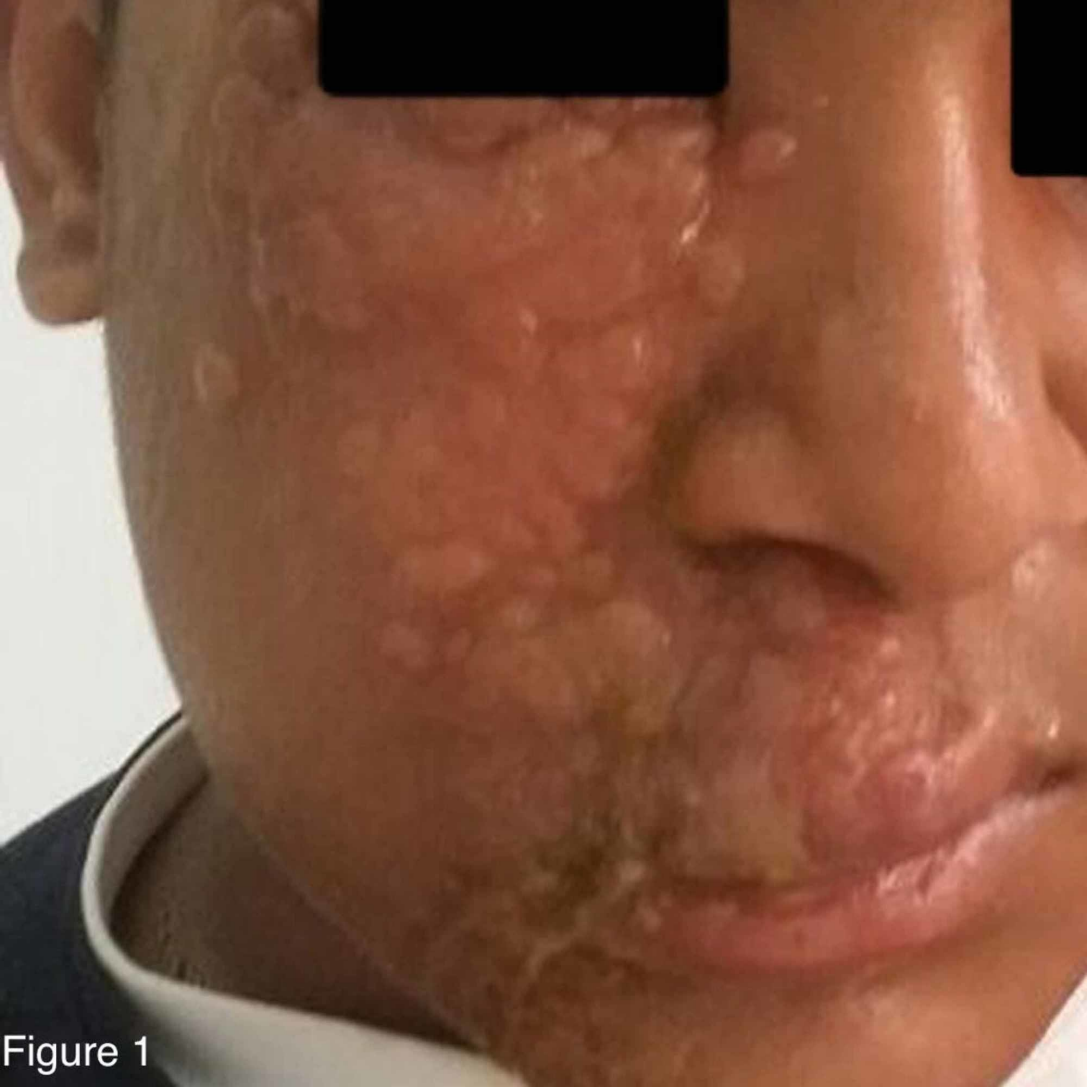
Multiple grouped vesicles on an erythematous base spreading over the right half of the face, following the maxillary nerve dermatomal distribution on the fifth day of admission

On examination on the fifth day after admission, the patient was conscious, oriented, not dehydrated, and not in distress. The rash appeared as multiple grouped vesicles on an erythematous base spreading over the right half of the face, following the maxillary nerve dermatomal distribution and sparing the forehead and chin (Figure 1). The patient’s mouth and right eye could not be examined as he was not able to open them due to pain. By the end of the first week after admission, ocular examination showed normal intraocular pressure and no redness of the sclera.

**Figure 2:**
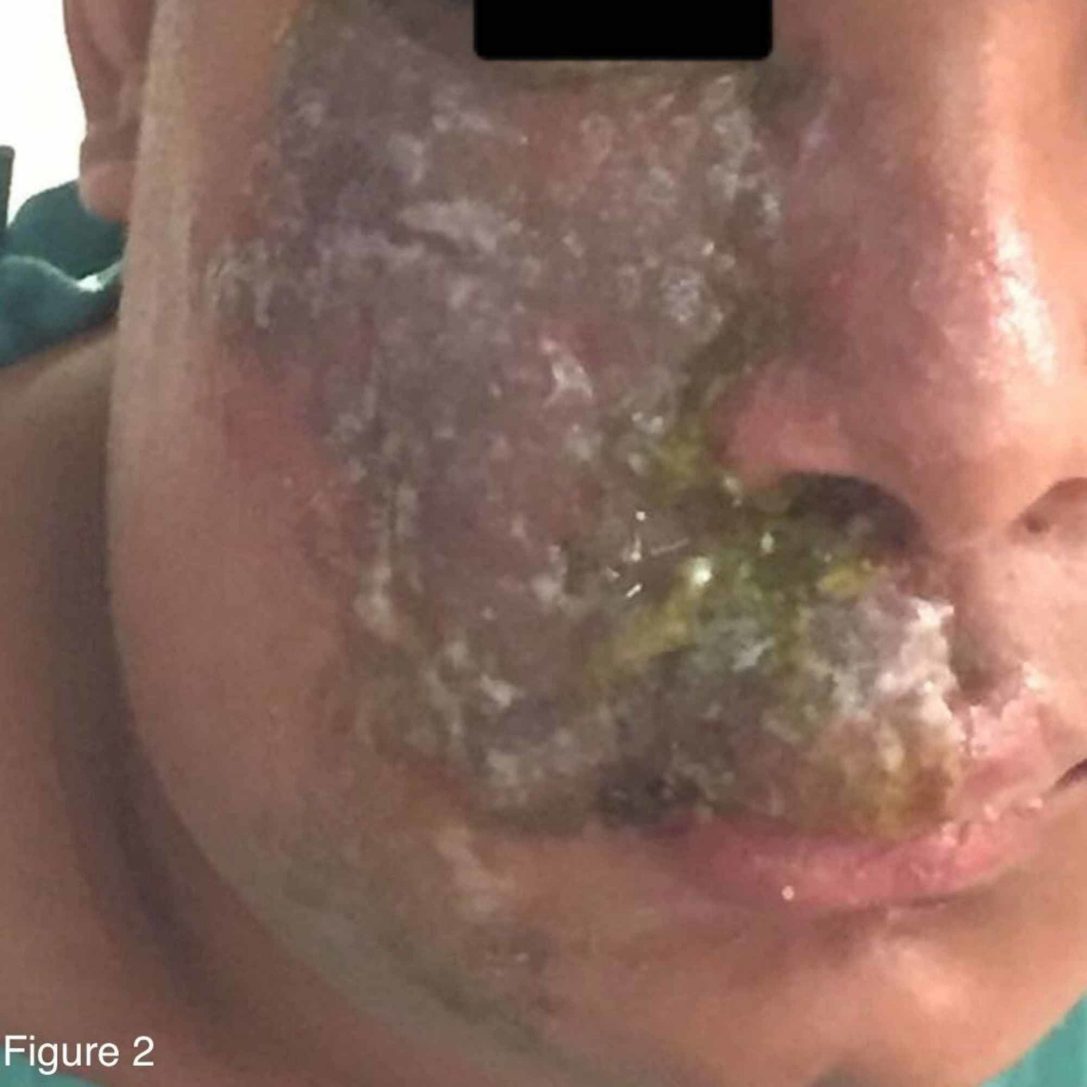
Crusting lesions with yellowish discoloration that resulted from ruptured bullae by the end of the first week of admission

**Figure 3:**
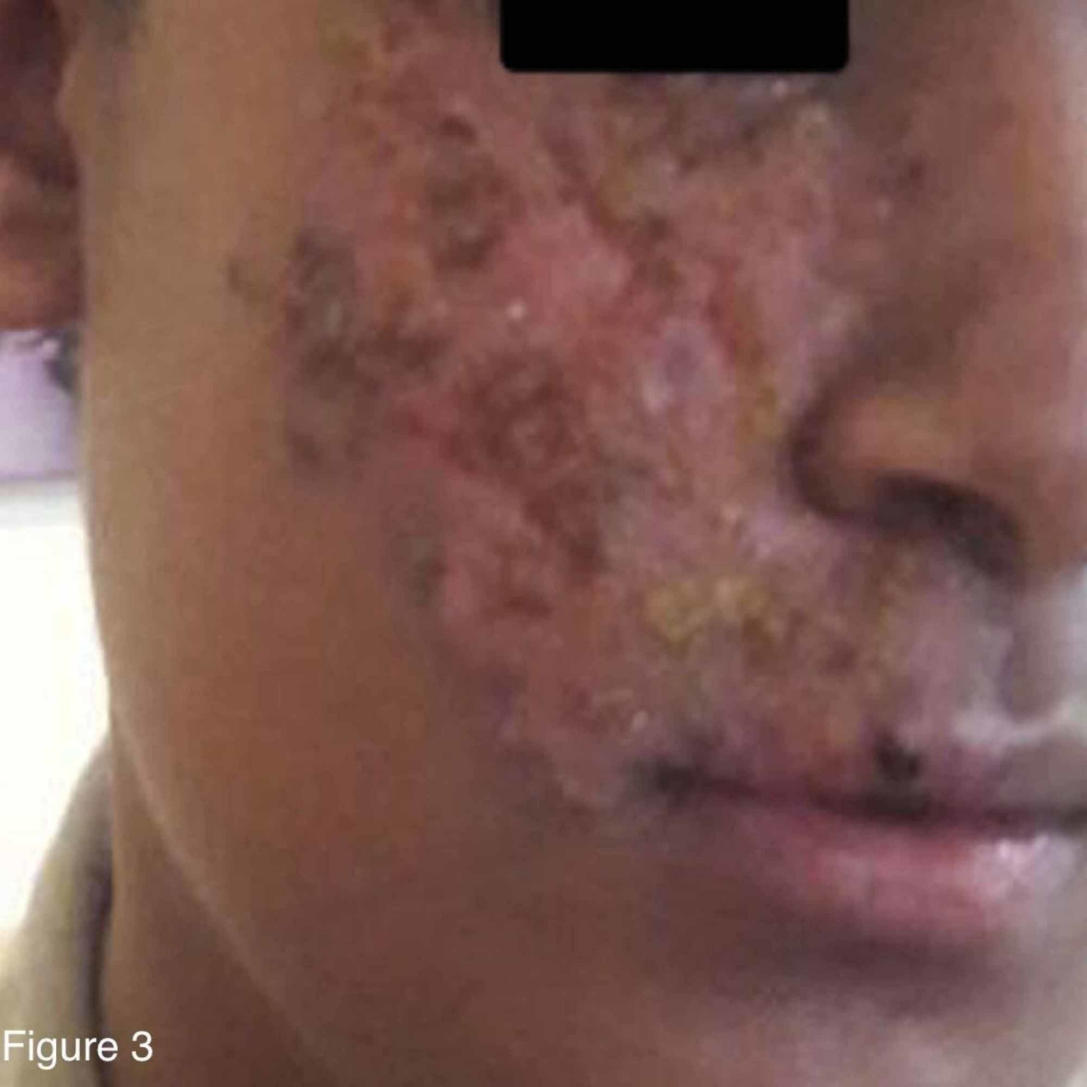
Painless lesions and peeling with healthy raw skin underneath by the second week from admission

The patient was admitted, with raised clinical suspicion of herpes zoster. Other differential diagnoses were zosteriform HSV infection, cellulitis, or an insect bite. The painful prodrome preceding the development of the vesicles was clinically correlated with herpes zoster. Zosteriform HSV infection was unlikely, as there was no history of recurrence and a blood test for HSV was negative. Other differential diagnoses were excluded clinically and by investigations. He was started on vancomycin and ceftriaxone. Acyclovir and clindamycin were added once the results of the skin biopsy were provided. He received vancomycin for two days, clindamycin for six days, acyclovir for seven days, and ceftriaxone for eight days. Thereafter, the result of the PCR returned and was positive for VZV. By the end of the first week, the vesicular rash had converted to bullae, which then ruptured to form crusting lesions with yellowish discoloration (Figure 2). For topical treatment of the crusting lesions, the patient was started on potassium permanganate and white petrolatum ointment (Vaseline) twice daily. By the second week, the patient’s overall condition had markedly improved (Figure 3). The lesions were painless, crusted, and peeling, with raw skin underneath, and no limitations of eye or mouth movement were observed. He was discharged on 800 mg acyclovir five times per day for seven days as well as topical potassium permanganate and Fucidin cream twice daily for one week. Ten days later, the patient was seen for follow-up in the outpatient clinic. The facial lesion continued to improve, and a follow-up appointment was arranged for two weeks later.

## Discussion

Reactivation of VZV infection in immunocompetent children is rare; fewer than 10% of zoster patients are younger than 20 years and only 5% are younger than 15 years [4]. A field study conducted in the UK showed an incidence of 0.74 per 1000 person-years among children aged zero to nine years and 1.38 per 1000 person-years among those aged 10-19 years [5]. It has been proposed that the reactivation of latent VZV is a consequence of decreased cell-mediated immunity as seen in immunocompromised and elderly individuals [6-7]. The reason for reactivation in healthy immunocompetent children remains unclear. Several studies have suggested that the risk factors for primary infection in children later presenting with herpes zoster include wild-type VZV infection, VZV vaccination, intrauterine exposure, penetrating trauma, emotional stress, and asthma [8-10]. In 2004, Thoms et al. reported that trauma was correlated with an increased risk of herpes zoster by approximately 12-folds. They reported that the risk is associated only at the site of trauma within one month of the skin rash development. These observations emphasize that a viral reactivation in the dorsal root ganglion of the nerve could be triggered by traumatic stimulation of the nerve [11]. In the current case, the patient may have been exposed to the primary infection in utero, through vaccination, or as a result of major facial trauma. However, the link to intrauterine exposure could not be confirmed due to the lack of proper documentation for the maternal varicella infection.

## Conclusions

In conclusion, herpes zoster results from the reactivation of a latent VZV infection and is rarely seen in healthy children; the reason for reactivation in this group remains unclear. A maternal VZV infection during pregnancy may play a role in the development of subsequent herpes zoster infection in the child. Moreover, herpes zoster can easily be confused with cellulitis or an insect bite, unless confirmed by laboratory and pathological investigations. Proper management is essential to prevent serious complications such as neurological sequelae.
